# Early discontinuation of adjuvant chemotherapy in patients with early-stage pancreatic cancer correlates with inferior survival: A multicenter population-based cohort study

**DOI:** 10.1371/journal.pone.0263250

**Published:** 2022-02-02

**Authors:** Javeria Muhammadzai, Kamal Haider, Michael Moser, Haji Chalchal, John Shaw, Donald Gardiner, Dorie-Anna Dueck, Osama Ahmed, Bryan Brunet, Mussawar Iqbal, Yigang Luo, Gavin Beck, Adnan Zaidi, Shahid Ahmed

**Affiliations:** 1 College of Medicine, University of Saskatchewan, Saskatoon, SK, Canada; 2 Medical Oncology, Saskatoon Cancer Centre, University of Saskatchewan, Saskatoon, SK, Canada; 3 Department of Surgery, University of Saskatchewan, Saskatoon, SK, Canada; 4 Medical Oncology, Allan Blair Cancer Centre, Regina, SK, Canada; 5 Radiation Oncology, Saskatoon Cancer Centre, University of Saskatchewan, Saskatoon, SK, Canada; Chang Gung Memorial Hospital at Linkou, TAIWAN

## Abstract

**Background:**

The current study aimed to determine the association between timing and completion of adjuvant chemotherapy and outcomes in real-world patients with early-stage pancreatic cancer.

**Methods:**

In this multi-center cohort study patients with early-stage pancreatic cancer who were diagnosed from 2007–2017 and underwent complete resection in the province of Saskatchewan were examined. Cox proportional multivariate analyses were performed for correlation with recurrence and survival.

**Results:**

Of 168 patients, 71 eligible patients with median age of 69 years and M:F of 37:34 were identified. Median time to the start of adjuvant therapy from surgery was 73 days. Of all patients, 49 (69%) patients completed adjuvant chemotherapy and 22 (31%) required early treatment discontinuation. Median recurrence-free survival of patients who completed treatment was 22 months (95%CI:15.8–28.2) vs. 9 months (3.3–14.7) if treatment was discontinued early (P<0.001). Median overall survival of those who completed treatment was 33 (17.5–48.5) vs. 16 months (17.5–48.5) with early treatment discontinuation (P<0.001). In the multivariate analysis, treatment discontinuation was significantly correlated with recurrent disease, hazard ratio (HR), 2.57 (1.41–4.68), P = 0.002 and inferior survival, HR, 2.55 (1.39–4.68), P = 0.003. No correlation between treatment timing and survival was noted.

**Conclusions:**

Early discontinuation but not the timing of adjuvant chemotherapy correlates with inferior outcomes.

## Introduction

Pancreatic cancer is a leading cause of cancer-related death. It is the 12^th^ most common cancer and 7^th^ most frequent cause of cancer-related death worldwide [1,2]. Each year, more than 450,000 patients are diagnosed with pancreatic cancer and about 430,000 patients die of it. In North America, it is the 4^th^ leading cause of cancer death among men and women [1–3]. Surgery is the primary curative option; however, only about 20% of patients have operable disease at the time of diagnosis [3–5]. Furthermore, the majority of patients who undergo surgery develop recurrent disease and die from it. Despite poor prognosis and a high risk of relapse, adjuvant chemotherapy after complete resection of the primary tumor has been associated with a significant reduction in the risk of recurrence and improvements in overall survival [6–13]. Based on the results of several randomized controlled trials, six months of adjuvant chemotherapy has become the standard of care and is recommended by the major international cancer organizations [4,5]. In the real-world setting, however, not all patients following surgery are optimal candidates for adjuvant chemotherapy. Furthermore, due to poor post-operative recovery after pancreatic cancer surgery, substantial delays in the commencement of adjuvant chemotherapy are not uncommon. Moreover, not all patients complete their planned adjuvant therapy. Although there is evidence in various solid cancers that a delay in the timing of adjuvant chemotherapy is associated with inferior outcomes, limited evidence for this is available in resected pancreatic cancer [14–17]. In addition, it is not well-studied if early discontinuation of planned adjuvant therapy in the real-world setting is associated with a higher risk of recurrent cancer and poorer survival. This multicenter study aimed to determine the association between the timing and completion of adjuvant therapy and outcomes in patients with early-stage adenocarcinoma of the pancreas who were diagnosed and treated with surgery followed by adjuvant chemotherapy in a Canadian province over a 10-year period. We hypothesized that both a delay in the initiation of adjuvant chemotherapy and early discontinuation of planned adjuvant systemic therapy are associated with inferior outcomes.

## Methods

### Study objectives and design

The University of Saskatchewan Biomedical Research Ethics Board approved the study protocol and waived informed consent (ID: 1149). The study objectives were to compare recurrence-free survival and overall survival in patients with completely resected pancreatic cancer who received adjuvant chemotherapy in relationship with the timing of adjuvant therapy and the completion of planned adjuvant therapy. It was a population-based multicenter retrospective cohort study.

### Eligibility criteria

Patients with adenocarcinoma of the pancreas who were diagnosed from January 2007 to December 2016, who underwent complete resection and received at least one treatment with adjuvant chemotherapy were included. Patients with other histopathologies such as neuroendocrine tumor, intrapapillary mucinous cystic neoplasms (IPMN), lymphoma or gastrointestinal stromal tumor (GIST) were excluded. Patients with metastatic disease at the time of diagnosis or who were found to have metastatic disease within three months of surgery were excluded. In addition, all patients with early-stage pancreatic cancer who did not receive adjuvant chemotherapy were excluded. International Classification of Disease (ICD) codes relevant for adenocarcinoma of the pancreas were used to identify eligible patients using the Saskatchewan Cancer Registry. The registry prospectively collects and updates the database. A pre-specified abstraction sheet was used for data collection and were abstracted by JZ. All patients were followed until the data cutoff of date of July 01, 2019.

### Definitions

Overall survival (OS) was defined as the time from the diagnosis of early-stage operable pancreatic cancer to death from any cause. Disease-free survival (DFS) was defined as the time from the surgery of early-stage operable pancreatic cancer to the date of recurrent disease, a new primary cancer or death from any cause. Recurrence-free survival was defined as the time from the surgery of early-stage operable pancreatic to the date of relapse of recurrent pancreatic cancer. Early discontinuation of adjuvant chemotherapy was defined as inability to receive all planned cycles of adjuvant chemotherapy regardless of the duration of treatment. The standard adjuvant chemotherapy regimens were gemcitabine for six cycles; gemcitabine in combination with capecitabine for six cycles; capectiabine for eight cycles and infusional 5FU, irinotecan, and oxaliplatin (FOLFIRINOX) for 12 cycles. The recommended duration of adjuvant therapy was 6 months. If adjuvant radiation therapy was recommended, it was administered in combination with 5FU or capcitabine after 4 months of chemotherapy.

### Analysis

The Kaplan-Meier survival method was used to estimate survival. Log rank tests were performed to compare the survival of different groups. Cox proportional multivariate analyses were performed to estimate if the timing of chemotherapy and completion of planned treatment independently correlate with survival. The hazard ratio and its 95% CI were estimated. The following variables were examined for their prognostic importance: age (65 vs. <65 years), gender, comorbid illness, World Health Organization (WHO) performance status (≥1 vs. <1), secondary cancer, T status (T3 vs. T1 or T2), grade (3 vs. 1 or 2), lymph node status, margin, carbohydrate antigen 19–9 (CA19-19) level (≥36 or <36 kU/L), neutrophil and lymphocyte ratio, post-operative complications, timing of adjuvant chemotherapy, surgery location, adjuvant radiation, single agent chemotherapy, and completion of planned treatment. In addition, for a parsimonious model and to avoid multicollinearity between T and nodal status a composite variable of unfavorable histopathology that was defined as presence of node positive disease or positive resection margin or T3 disease was examined. Following the univariate analysis, all the variables that were found to have P-value ≤0.25 were fitted in a multivariate model, to examine their correlation with survival. The likelihood ratio test and *t* test were used to assess if adding an independent variables of interest added significantly to the prediction of survival in the model. A two-sided P-value of <0.05 was considered to be statistically significant. SPSS version 24.0 (IBM, Armonk, NY) was used for statistical analysis.

## Results

**Fig 1:**
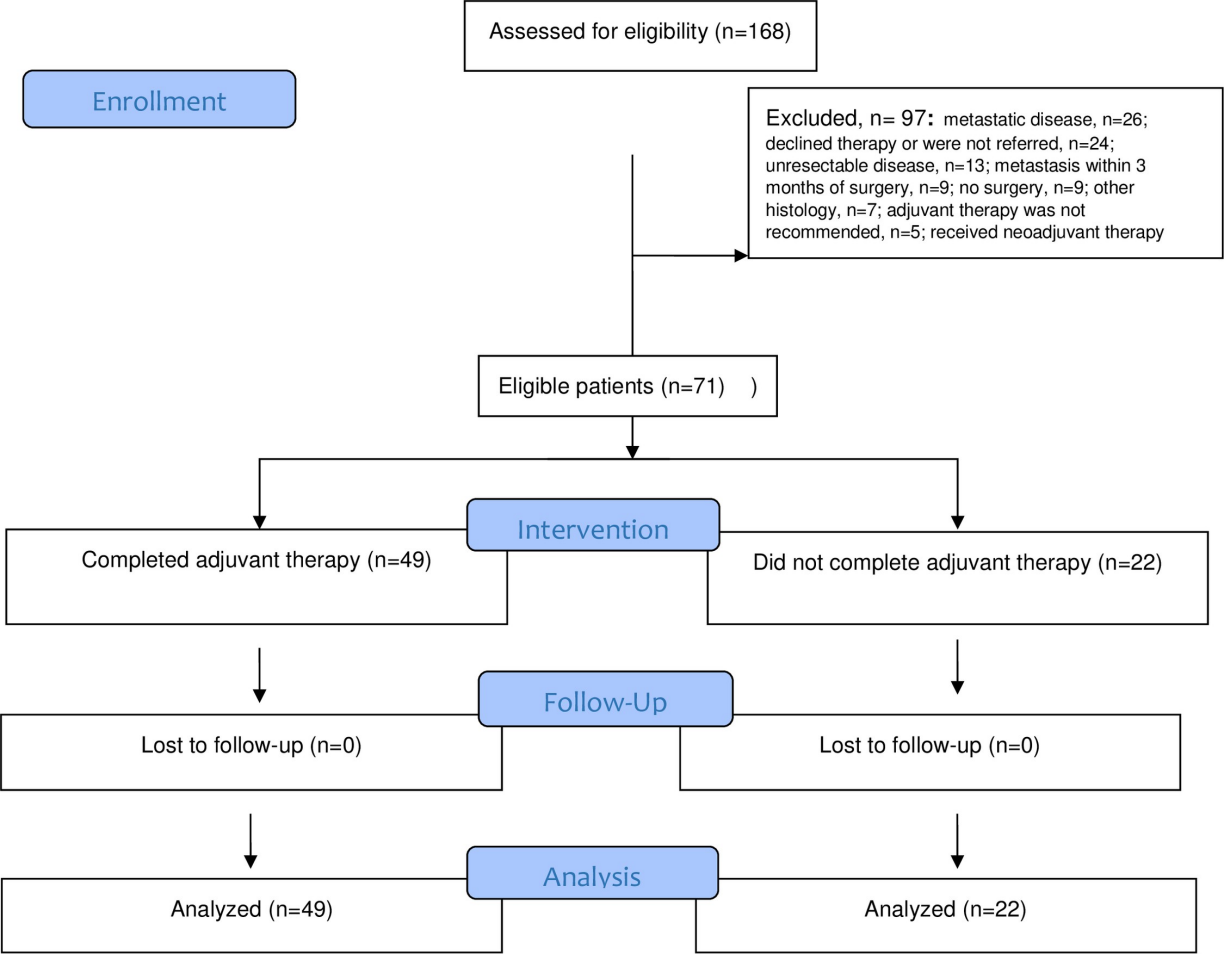
Flow diagram of eligible patients with pancreatic cancer eligible for the study.

Using ICD code 168, patients with pancreatic cancer were identified. Of those, 97 patients were not found to be eligible (**Fig 1**). Metastatic or locally inoperable advanced disease (n = 39) and lack of surgery or adjuvant chemotherapy (n = 38) were the most common reasons for exclusion. Overall, 71 eligible patients were identified. Patient characteristics are described in **Table 1**. The median age of the entire cohort was 69 years (interquartile range [IQR]: 57–73) with a male to female ratio of 37:34. Overall, 56 (79%) patients had a WHO performance status of 0 or 1, 92% had a major comorbid illness, 42% of patients had underlying diabetes mellitus and 24% of patients were active smokers at the time of diagnosis. The majority of patients (77%) had pancreatic head tumors with a median tumor size of 3 cm. Of 71 patients, 55 (78%) underwent Whipple’s procedure (**Table 2**). Of all patients Median time to the start of adjuvant chemotherapy from surgery was 73 days (IQR: 59–89); 32% started adjuvant chemotherapy within 60 days of surgery; 89% received single agent chemotherapy, mostly gemcitabine, and 25% received adjuvant radiation. Of all patients, 49 (69%) patients completed planned 6 months of adjuvant chemotherapy with or without adjuvant radiation and 22 (31%) patients required early treatment discontinuation. No significant differences were noted between the two groups in relation to baseline characteristics. However, patients who did not complete adjuvant chemotherapy had significantly higher level of baseline CA19-19 level. The reasons of early discontinuation of adjuvant therapy are described in **Table 3**. Majority of patients stopped treatment due to the treatment-related side effects. Median duration of hospital stay following pancreatic surgery was 14 days (IQR: 10–23), 13 days in patients who completed adjuvant therapy vs. 22 days in patients who did not complete treatment (p = 0.01). Overall, 73% of patients who did not complete adjuvant therapy experienced post-operative complications compared to 35% of patients who completed the planned adjuvant therapy. Median time to the initiation of adjuvant chemotherapy was 73 days; the time was significantly longer in patients with required early discontinuation of adjuvant therapy (76 days vs. 69, p = 0.028). Patients who did not complete adjuvant chemotherapy received a mean 2.9±1.3 cycles of adjuvant chemotherapy compared to 6.2±1.2 cycles in patients who completed planned treatment. Likewise, median duration of chemotherapy of 2.5 months in patients who did not complete adjuvant therapy was significantly shorter compared to 6 months in patients who completed planned treatment.

**Table 1:** Patients baseline characteristics prior to the commencement of adjuvant therapy.

Variables	All N = 71 (%)	Completed Planned Treatment N = 49 (%)	Early discontinuation of Adjuvant therapy N = 22 (%)	P values
Age	69 (IQR 57–73)	68 (IQR: 59–73)	70 (IQR 56–73)	0.90
Age ≥65 years	41 (58)	29 (59)	12 (59)	0.79
Male Sex	37 (52)	24 (49)	13 (59)	0.45
WHO Performance Status (%)				
0	22 (31)	18 (37)	4 (18)	0.16
1	34 (48)	22 (45)	12 (55)	0.60
2	12 (17)	6 (12)	6 (27)	0.17
3	3 (4)	3 (6)	0	0.54
Major Comorbid illness	65 (92)	43 (88)	22 (100)	0.16
Diabetes Mellitus	30 (42)	20 (41)	10 (46)	0.79
Current Smoking	12 (17)	6 (12)	6 (27)	0.17
Secondary Cancer	17 (24)	12 (25)	5 (23)	1.0
Tumor Location				
Head	55 (77)	38 (78)	17 (77)	1.0
Body	6 (9)	4 (8)	2 (9)	1.0
Tail	10 (14)	7 (14)	3 (14)	1.0
Median size (cm)	3 (IQR 2–4)	2.9 (IQR 2.5–4)	3.1 (IQR 2.5–3.8)	0.61
Positive margin	15 (21)	12 (25)	3 (14)	0.36
T Status				
T1	3 (4)	1 (2)	2 (9)	0.22
T2	21 (30)	17 (35)	4 (18)	0.26
T3	47 (66)	31 (63)	16 (72)	0.58
Node positive	45 (63)	32 (65)	13 (59)	0.79
Unfavorable histopathology	60 (85)	41 (84)	19 (86)	1.0
Stage				
IA	1 (1.4)	0	1 (5)	0.30
IB	11 (16)	9 (18)	2 (9)	0.48
IIA	14 (20)	8 (16)	6 (27)	0.34
IIB	45 (63)	32 (65)	13 (59)	0.79
Creatinine*	73 ± 25	74.5 ± 27	70 ± 21	0.48
Urea*	4.9 ± 1.9	4.9 ± 1.9	5.0 ± 2.1	0.85
Albumin*	34 ± 4.6	35 ± 4.3	33.5± 5.4	0.37
Bilirubin*	9.1 ± 6.7	8.3 ± 4.4	11.1 ± 10.3	0.12
Alkaline Phosphatase*	151 ± 110	131 ± 96	200 ± 130	0.02
ALT*	41 ± 56	35 ± 32	54 ± 91	0.21
LDH*	147 ± 30	142 ± 18	154 ± 45	0.42
WBC*	8.2 ± 2.9	7.7 ± 2.4	9.2 ± 3.5	0.04
Hemoglobin*	121 ± 16	120 ± 14	121 ± 20	0.83
Platelet*	340 ± 165	329 ± 109	365 ± 249	0.39
Median Neutrophil: Lymphocyte	2.5 (IQR 1.6–3.7)	2.8 (IQR 1.6–3.6)	2.4 (IQR 1.5–4.6)	0.68
Median CA19-9	22 (IQR 13–105)	20 (IQR 10–70)	37 (IQR13-253)	0.025

*Mean laboratory values and standard deviations (±) are provided; ALT: Alanine transaminase; CA19-9: Carbohydrate antigen 19–9; IQR: Interquartile range; LDH: Lactate dehydrogenase; WBC: White blood cell; WHO: World Health Organization.

**Table 2:** Interventions and various outcomes in the two groups of patients with early stage pancreatic cancer.

Variables	All N = 71 (%)	Completed Planned Treatment N = 49 (%)	Early discontinuation of Adjuvant therapy N = 22 (%)	P values
Type of Surgery				
Whipple Procedure	55 (78)	38 (78)	17 (77)	1.0
Distal Pancreatectomy	16 (22)	11 (22)	5 (23)	1.0
Median hospital stay (days)	14 (IQR 10–23)	13 (IQR 10–21)	22 (IQR 11–28)	0.01
Post-operative complications*	33 (47)	17 (35)	16 (73)	0.004
Wound infection	13 (18)	8 (16)	5 (23)	0.52
Other infection	12 (17)	6 (12)	6 (27)	0.17
Anastomotic leak	7 (10)	4 (8)	3 (14)	0.66
Thromboembolism	5 (7)	1 (2)	4 (18)	0.02
Others	13 (18)	4 (8)	9 (41)	0.002
Median Time to Adjuvant Therapy (days)	73 (IQR: 59–89)	69 (IQR: 57–86)	76 (IQR 66–98)	0.028
Chemotherapy Regimen				
Gemcitabine	62 (87)	42 (86)	20 (91)	0.71
Capecitabine	1 (1.4)	1 (2)	0	1.0
Gem/Cap	6 (8.5)	4 (8)	2 (9)	1.0
FOLFIRINOX	2 (2.8)	2 (4)	0	1.0
Mean Cycles	5.2±6.0	6.2 ± 1.2	2.9 ± 1.3	<0.001
Median Chemotherapy Duration (months)	4.4 (IQR: 3.5–6)	6 (IQR: 5–6)	2.5 (IQR: 1–3)	<0.001
Adjuvant Radiation	18 (25)	15 (31)	3 (14)	0.15
Anticoagulant	18 (25)	10 (20)	8 (36)	0.23
Recurrent Cancer	52 (73)	32 (65)	20 (91)	0.04
Received Palliative Chemotherapy	23 (32)	17 (53)	6 (30)	0.15

*were not mutually exclusive; IQR: Interquartile range.

**Table 3:** The reasons of early discontinuation of adjuvant chemotherapy.

Reasons	Number 22 (%)
Cardio-vascular complications including stroke, myocardial infarction or heart failure	3 (14)
Delayed post-operative complications	2 (9)
Treatment-related toxicities	
Abnormal liver function	2 (9)
Infection	4 (18)
Lung toxicity	1(4)
Gastrointestinal toxicity	2 (9)
Fatigue and asthenia	3 (14)
Non-compliant	1 (4)
Declined by patient	1 (4)
Suspected recurrent disease	3 (14)

### Survival

**Fig 2:**
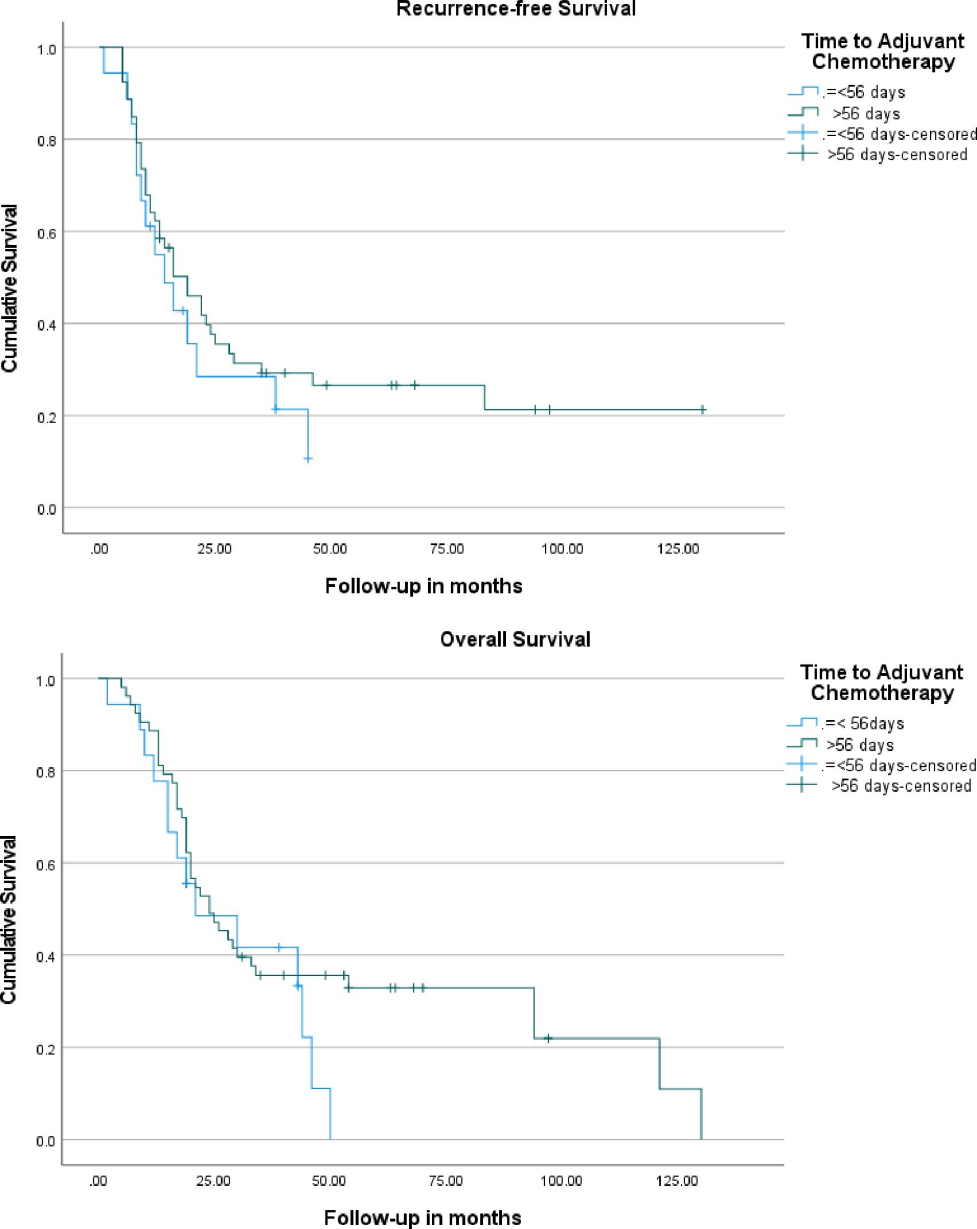
A. Kaplan-Meier recurrence-free survival curves of the patients who received adjuvant chemotherapy within 56 days of surgery to those who received adjuvant therapy beyond 56 days. B. Kaplan-Meier overall survival curves of the patients who received adjuvant chemotherapy within 56 days of surgery to those who received adjuvant therapy beyond 56 days.

The total follow up duration was 138 months and median follow up period was 22 months. Median recurrence-free and overall survival of entire cohort were 16 months (95% CI: 11.0–21.0) and 24 months (95% CI: 16–32.0), respectively. Median recurrence-free survival of the patients who received adjuvant chemotherapy within 56 days of surgery was 14 months (95%CI: 6.3–21.7) compared to 19 months (95% CI: 11.5–26.5) in patients who received adjuvant treatment beyond 56 days of surgery (p = 0.34) [**Fig 2A**]. The median OS of the patients who received adjuvant chemotherapy within 56 days of surgery was 21 months (95% CI: 2.2–39.8) compared to 24 months (95% CI: 15.8–32.2) in patients who received adjuvant treatment beyond 56 days of surgery (p = 0.24) [**Fig 2B**].

**Fig 3:**
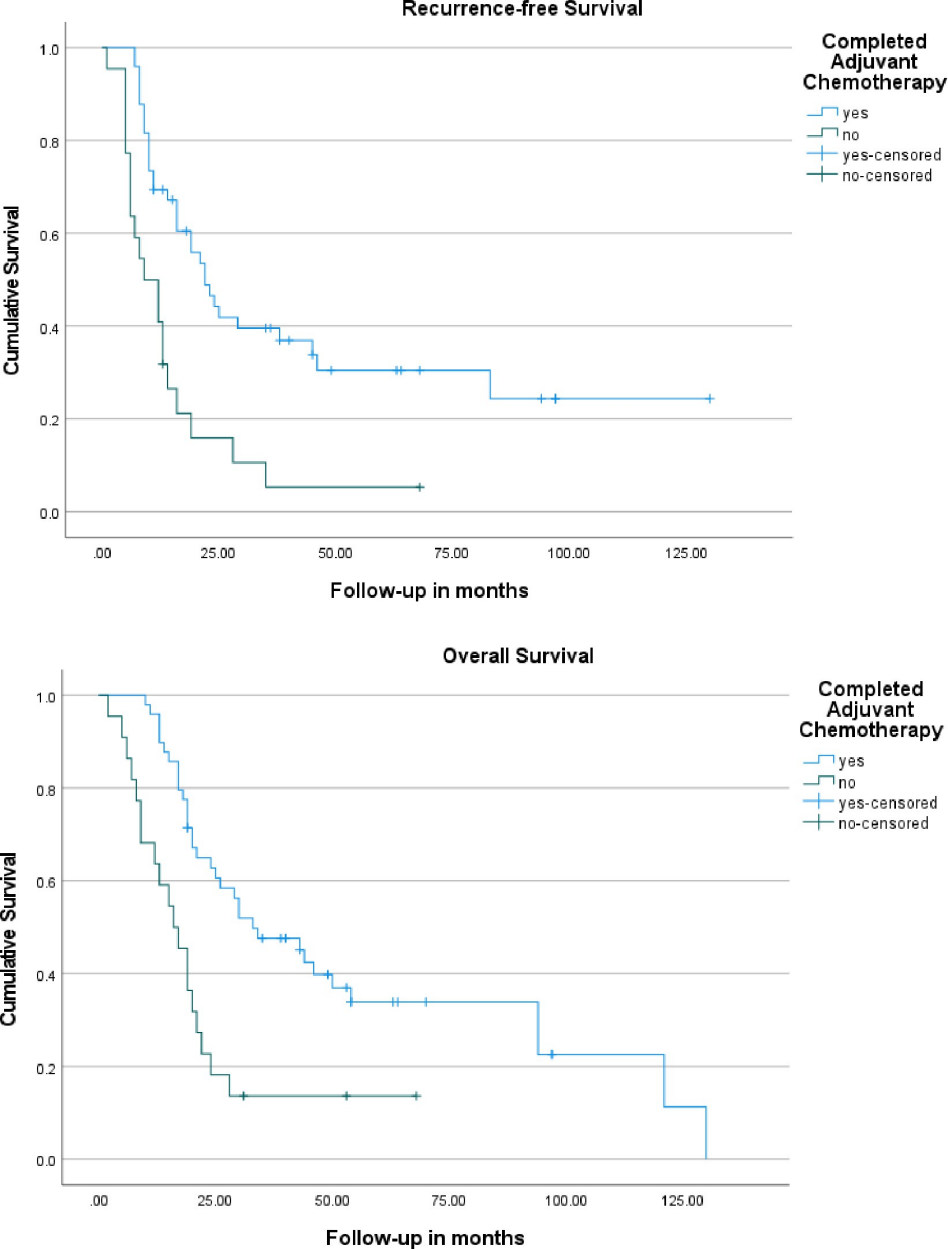
A. Kaplan-Meier recurrence-free survival curves of the patients who completed adjuvant chemotherapy to those who did not complete adjuvant therapy. B. Kaplan-Meier overall survival curves of patients who received planned adjuvant chemotherapy to those did not complete adjuvant therapy.

The median recurrence-free survival of patients who completed treatment was 22 months (95%CI: 15.8–28.2) vs. 9 months (3.3–14.7) if treatment was discontinued early (P<0.0001) [**Fig 3A**]. The median DFS of patients who completed planned treatment was 21 months (95% CI: 14.4–27.6) compared to 8 months (95% CI: 3.4–12.6) if they did not complete adjuvant therapy (P<0.0001). Median overall survival of the entire cohort was 24 months (95%CI: 16.1–31.9). Median overall survival of the patients who completed planned treatment was 33 months (10.5–21.5) compared to 16 months (17.5–48.5) if it was stopped early (P<0.0001) [**Fig 3B**].

Patients who completed planned treatment had one-year, two-year, three-year, and five-year OS rates of 89%, 63%, 48% and 34%, compared to 63%, 18%, 14% and 14%, respectively for those who did not complete adjuvant chemotherapy.

### Cox proportional multivariate analysis

In the univariate analysis, only early discontinuation of adjuvant therapy was significantly correlated with recurrent disease, HR 2.85 (95%CI: 1.59–5.02), P = 0.001. In the final model, early discontinuation of adjuvant therapy was examined with WHO performance status of ≥1, and unfavorable histopathology that was defined as presence of one of the factor; T3 disease, node positive cancer and positive margin. The multivariate model showed that early discontinuation of treatment was independently correlated with survival, HR for RFS 2.57 (95% CI: 1.41–4.68), p = 0.002 (**Table 4**). With respect to survival, in the univariate analysis, only early discontinuation of adjuvant therapy significantly correlated with a higher risk of mortality, HR for OS 2.78 (1.55–5.0), p = 0.001. In the final multivariate model, after adjustment for low performance status, and unfavorable histopathology, failure to complete adjuvant chemotherapy significantly correlated with inferior survival, HR for OS 2.55 (1.39–4.68), P = 0.003 (**Table 5**). No correlation between timing of adjuvant therapy and survival was noted. On a secondary exploratory analysis after exclusion of 3 cases of suspected recurrence during adjuvant chemotherapy the adjusted hazard ratios for RFS and OS were 2.40 (1.30–4.50), p = 0.006 and 2.35 (1.25–4.42), p = 0.008, respectively.

**Table 4:** Cox proportional analysis and modelling of various clinical and pathological variables that correlate with recurrence of the disease (Recurrence-free survival).

Variable	HR (95% CI) Univariate	P value	HR (95% CI) Multivariate	P value
Age	0.96 (0.55–1.66)	0.89	-	-
WHO Performance Status ≥1	1.50 (0.80–2.74)	0.20	1.21 (0.63–2.30)	0.58
Comorbid illness	1.41 (0.44–4.54)	0.54	-	-
Male Sex	0.80 (0.46–1.38)	0.42	-	-
Secondary Cancer	0.94 (0.49–1.81)	0.85	-	-
T3 tumor	1.40 (0.77–2.53)	0.25	-	-
Node positive	1.57 (0.87–2.84)	0.12	-	-
Positive margin	1.5 (0.79–2.8)	0.22	-	-
Time to chemotherapy	1.0 (0.99–1.01)	0.91	-	-
Neutrophil: lymphocyte	0.96 (0.82–1.13)	0.62	-	-
Postoperative complication	1.14 (0.66–1.96)	0.62	-	-
CA19-9 >36 kU/ml	1.37 (0.80–2.37)	0.26	-	-
Location of surgery	0.99 (0.55–1.68)	0.90	-	-
Adjuvant Radiation	0.78 (0.41–1.46)	0.42	-	-
Single agent chemotherapy	1.28 (0.46–3.58)	0.61	-	-
Unfavorable histopathology	1.97 (0.84–4.62)	0.11	1.79 (0.76–4.2)	0.18
Early discontinuation of adjuvant treatment	2.85 (1.59–5.02)	0.001	2.57 (1.41–4.68)	0.002

**Table 5:** Cox proportional analysis and modelling of various clinical and pathological variables that correlate with mortality (Overall survival).

Variable	HR (95% CI) Univariate analysis	P value	HR (95% CI) Multivariate analysis	P value
Age	1.33 (0.76–2.34)	0.32	-	-
WHO Performance Status >2	1.47 (0.78–2.76)	0.22	1.23 (0.64–2.36)	0.52
Comorbid illness	1.23 (0.38–3.96)	0.72	-	-
Male Sex	0.83 (0.48–1.45)	0.53	-	-
Secondary Cancer	0.74 (0.38–1.45)	0.37	-	-
T3 tumor	1.23 (0.68–2.22)	0.49	-	-
Node positive	1.60 (0.87–2.86)	0.12	-	-
Positive margin	1.5 (0.83–2.80)	0.19	-	-
Time to chemotherapy	1.0 (0.99–1.0)	0.61	-	-
Neutrophil: lymphocyte ≥2.5	0.99 (0.85–1.16)	0.98	-	-
Postoperative complication	1.18 (0.69–2.05)	0.55	-	-
CA19-9 >36 kU/ml	1.24 (0.71–2.20)	0.45	-	-
Adjuvant Radiation	1.02 (0.56–1.86)	0.94	-	-
Single agent chemotherapy	1.90 (0.58–6.0)	0.30	-	-
Unfavorable histopathology	1.71 (0.73–4.02)	0.21	1.52 (0.64–3.60)	0.33
Early discontinuation of adjuvant treatment	2.78 (1.55–5.0)	0.001	2.55 (1.39–4.68)	0.003

## Discussion

Surgical resection followed by adjuvant chemotherapy is the current standard treatment for patients with early-stage pancreatic cancer and confers substantial survival benefit. Our results suggest that, in a real-world setting, early discontinuation of adjuvant chemotherapy correlates with inferior survival. However, a delay in the initiation of chemotherapy was not associated with inferior outcomes. It is important to note that only about 30% patients started adjuvant chemotherapy within 8 weeks of surgery. About 60% patients of the study cohort were 65 years or older with median age of 69 years, and most patients had an underlying comorbid illness. Patients who were not able to complete their treatment had a high rate of post-operative complication and significantly longer duration of hospital stay, resulting in a delay in the initiation of adjuvant chemotherapy. However, after adjustment for age, nodal status, margin, post-operative complications, timing of adjuvant therapy and other variables, early discontinuation of adjuvant chemotherapy was independently associated with inferior outcomes. Overall patients who were not able to complete the planned adjuvant chemotherapy had almost a three-fold higher risk of recurrence and death compared to those who completed adjuvant chemotherapy. Our results are in agreement with the European Study Group for Pancreatic Cancer-3 (ESPAC-3) trial [18]. In this trial, 985 patients with early-stage pancreatic cancer were randomized to six cycles of adjuvant gemcitabine or to 5-FU and folinic acid. Of all patients, 68% completed all six cycles of intended therapy. A post-hoc analysis of the ESPAC-3 trial showed that the median survival of patients who completed the planned treatment was 33 months compared to 14.6 months if they discontinued adjuvant therapy earlier [18]. Likewise, the median recurrence-free survival of patients who received all six cycles was 16.5 months compared to 8.9 months if they did not complete adjuvant chemotherapy. However, similar to our findings, the timing of initiation of adjuvant chemotherapy was not an independent prognostic factor. A recent systematic review and meta-analysis of six studies involving 2031 patients with early-stage pancreatic cancer examined the correlation between the timing of adjuvant chemotherapy and survival and concluded that early commencement of adjuvant chemotherapy was not associated with better survival [14]. Another study using the National Cancer Database for the period 2003–2011 found no difference in the survival of patients who received adjuvant chemotherapy within 12 weeks of pancreatic surgery versus those who received adjuvant treatment beyond 12 weeks of surgery [19]. Although it is desirable to start adjuvant chemotherapy soon after surgery, a large number of patients following Whipple’s procedure develop complications, resulting in a delay in the initiation of adjuvant therapy. It is also reflected by the median time to the start of adjuvant therapy of 73 days or about 10 weeks in the entire cohort. Hence, in the setting of post-operative complications or slow recovery, it is not unreasonable to wait to start adjuvant therapy until patients recover from surgery to avoid early discontinuation of treatment and maximize the chance of completing adjuvant chemotherapy. Furthermore, optimal management of comorbid illnesses such as diabetes mellitus is important in patients with pancreatic cancer as an underlying comorbid illness may not only affect the recovery from pancreatic surgery, but may also increase the risk of adverse events related to adjuvant therapy [20].

It is important to highlight some limitations of the current study. The key limitation of the study is that this was a retrospective study with a relatively small sample size. Furthermore, only 10% of patients received combination chemotherapy, which is the current standard for patients with early-stage pancreatic cancer who are candidates for combination chemotherapy. Hence, it is not known if the study findings can be generalized to patients who are treated with combination chemotherapy. On the other hand, this study reviewed the outcomes of a whole Canadian province over a 10-year period with no selection bias, which is one of the major strengths of this study. In addition, patients who were found to have metastatic disease within three months of surgery were excluded to eliminate a potential bias of having patients with metastatic disease.

In summary, this well-designed population-based retrospective cohort study suggests that early discontinuation but not the timing of adjuvant chemotherapy in patients with early-stage pancreatic cancer has been associated with a higher risk of recurrent disease and inferior survival. It is unlikely that this question will be examined in the setting of a randomized control trial. Given that adjuvant chemotherapy has been associated with a substantial survival benefit in patients with early-stage pancreatic cancer, it is reasonable to consider it even if there is more than a 12-week delay following surgery. Likewise, it is important to complete adjuvant chemotherapy to avoid detrimental outcomes.

## Ethics

This study was approved by the University of Saskatchewan’s Biomedical Research Ethic Board. Participant consent was waived due to it being impossible or impractical to seek individual consent. The use of participant information without their consent is unlikely to adversely affect their welfare. Investigators have taken appropriate measure to safely protect the individuals and their personal information. Data collection and statistical analysis did not involve direct or indirect patient contact. Data was collected and deidentified, and only aggregate data is being published.
